# On the Origin of the Surprisingly Sluggish Redox Reaction of the N2O/CO Couple Mediated by [Y2O2]+C and [YAlO2]+C Cluster Ions in the Gas Phase**

**DOI:** 10.1002/anie.201208559

**Published:** 2012-12-06

**Authors:** Jia-Bi Ma, Zhe-Chen Wang, Maria Schlangen, Sheng-Gui He, Helmut Schwarz

**Affiliations:** a Institut für Chemie der Technischen Universität BerlinStrasse des 17. Juni 115, 10623 Berlin (Germany); b Chemistry Department, Faculty of Science, King Abdulaziz Universityeddah 21589 (Saudi Arabia); c Department of Chemistry, Colorado State UniversityFort Collins, CO 80526 (USA); d State Key Laboratory for Structural Chemistry of Unstable and Stable Species, Institute of Chemistry, Chinese Academy of Sciences100190 Beijing (People's Republic of China)

**Keywords:** cluster ions, density functional caclculations, gas-phase reactions, homogeneous catalysis, ion–molecule reactions

Catalytic conversion of harmful gases produced in fossil-fuel combustion or in large-scale chemical transformations, such as CO or the oxides of nitrogen into nitrogen and carbon dioxide, is of utmost importance both environmentally and economically. For example, N_2_O is a potent greenhouse gas with a warming potential exceeding that of CO_2_ by a factor of 300,^1^ and its role in the depletion of stratospheric ozone is well known.^2^ While these redox reactions are exothermic, for example Δ_r_*H*=−357 kJ mol^−1^ for the process N_2_O + CO→N_2_ + CO_2_, they do not occur directly to any measurable extent at either room or elevated temperatures because of high energy barriers that exceed the 193 kJ mol^−1^ for the N_2_O/CO couple. Catalysts are required to open-up new, energetically more favorable pathways,^3^ and the first example of a homogeneous catalysis in the gas phase, whereby atomic transition-metal cations bring about the efficient reduction of N_2_O by CO, was reported in a landmark study by Kappes and Staley,^4^ which was followed in the ensuing decades by numerous investigations.^5^ Recently, these studies addressed more specific questions, for example, “catalyst poisoning”, and these experiments revealed remarkable effects of both the cluster size and the charge state of the catalysts.^6^ For example, the active species of the Pt_7_^+^ cluster are Pt_7_^+^, [Pt_7_O]^+^, [Pt_7_O_2_]^+^, and [Pt_7_(CO)]^+^ and it has a turnover number >500 at room temperature. The adsorption of more than one CO molecule onto the Pt_7_^+^ cluster, however, completely quenches the catalytic activity. Thus, coverage effects for any cluster sizes can be studied at a strictly molecular level. Similarly, the concept of “single-site catalysts”,^7^ the proper characterization and identification of which constitutes one of the challenges and intellectual cornerstones in contemporary catalysis, can be probed directly in gas-phase experiments with mass-selected heteronuclear metal-oxide clusters. For example, catalytic room-temperature oxidation of CO by N_2_O can be mediated by the bimetallic oxide cluster couple [AlVO_4_]^+.^/[AlVO_3_]^+.^.^8^ In the presence of CO, the cluster ion [AlVO_4_]^+.^ is efficiently reduced to [AlVO_3_]^+.^, and if N_2_O is added, the reverse reaction occurs. Both processes are clean and proceed with efficiencies (*ϕ*) of 59 % and 65 %, respectively, relative to the collision rates. Most interestingly, the two redox reactions occur at the Al-O_t_^.^ unit of the cluster (O_t_: terminal oxygen atom); bond activation involving the V—O moiety cannot compete kinetically and thermochemically. Thus, the existence and operation of an “active site” of a catalyst can already be demonstrated in a rather small heteronuclear cluster.^9^

In view of the intriguing role of, for example, doping effects in the gas-phase reactions of heteronuclear cluster oxides,9b,e,f the investigation of the [YAlO_2_]^+.^/[YAlO_3_]^+.^ and [Y_2_O_2_]^+.^/[Y_2_O_3_]^+.^ couples in the context of CO/N_2_O conversion deemed interesting, and herein we report our rather unexpected experimental/computational findings.

As shown in Figure 1 a, mass-selected, thermalized [YAlO_2_]^+.^ ions can be converted by N_2_O into [YAlO_3_]^+.^ with a bimolecular rate coefficient of 9.1×10^−12^ cm^3^ s^−1^ molecule^−1^, which amounts to *ϕ*=1 % relative to the collision rate.^10^ The by-product [YAlO_3_H]^+^ arises from hydrogen-atom transfer (HAT)^11^ from background impurities such as water or residual hydrocarbons. If a mixture of CO and N_2_O (1:19) is introduced into the reaction cell, the ion-intensity ratio [YAlO_3_]^+.^/[YAlO_2_]^+.^ decreases (Figure 1 b) compared to that shown in Figure 1 a. Clearly, reduction of [YAlO_3_]^+.^ occurs in the presence of CO; thus, a full thermal catalytic cycle is in operation at room temperature. The time evolutions of the reactions of [YAlO_2_]^+.^ in the presence of pure N_2_O and with a mixture of CO and N_2_O are shown in Figure 1 e,f, respectively. Clearly, the depletion of the signal for [YAlO_2_]^+.^ is retarded in the presence of CO because of reduction of the transiently formed [YAlO_3_]^+.^ cluster ion. By fitting the experimental data of the reaction sequence [YAlO_2_]^+.^⇄[YAlO_3_]^+.^→[YAlO_3_H]^+^,^12^ one can estimate the bimolecular rate coefficient for the conversion [YAlO_3_]^+.^→[YAlO_2_]^+.^ to be 5.3×10^−10^ cm^3^ s^−1^ molecule^−1^ (*ϕ*=77 %). As a consequence of the rather low intensity of the [YAlO_3_]^+.^ cluster ion, generated from [YAlO_2_]^+.^, a direct determination of the reaction rate for the process [YAlO_3_]^+.^ + CO→[YAlO_2_]^+.^ + CO_2_ was not possible.

**Figure 1:**
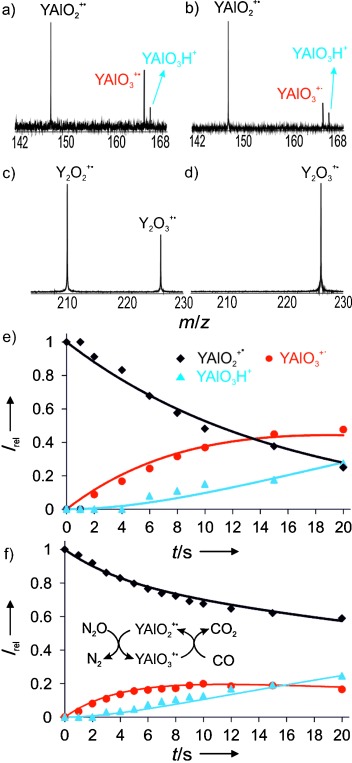
Mass spectra showing the reactions of a) [YAlO_2_]^+.^ with pure N_2_O (at a pressure of 1.9×10^−7^ mbar) after a reaction time of 8 s; b) [YAlO_2_]^+.^ with a 1:19 mixture of CO (at a pressure of 1.0×10^−8^ mbar) and N_2_O (1.9×10^−7^ mbar) after a reaction time of 8 s; c) [Y_2_O_2_]^+.^ with N_2_O at a pressure of 5.8×10^−9^ mbar after a reaction time of 5 s; d) [Y_2_O_3_]^+.^ with CO at a pressure of 1.0×10^−7^ mbar after a reaction time of 10 s. Kinetic fits of the reactions of [YAlO_2_]^+.^ with pure N_2_O and a 1:19 mixture of CO and N_2_O are shown in (e) and (f), respectively. The inset in (f) depicts the catalytic redox cycle.

As shown in Figure 1 c, the homonuclear cluster ion [Y_2_O_2_]^+.^ can also be oxidized by N_2_O to [Y_2_O_3_]^+.^, and the bimolecular rate coefficient amounts to 4.2×10^−10^ cm^3^ s^−1^ molecule^−1^ (*ϕ*=60 %). However, in sharp contrast to the [YAlO_3_]^+.^/CO couple, reduction of [Y_2_O_3_]^+.^ with CO does not occur at a measurable rate (Figure 1 d), even at a relatively high pressure of CO (up to 1×10^−7^ mbar) and a rather long reaction time (up to 10 s).

To obtain insight into the origins of the amazingly different reactivities of the [Y_2_O_*n*_]^+.^ and [YAlO_*n*_]^+.^ redox systems (*n*=2, 3) towards N_2_O and CO, DFT calculations using the B3LYP functional were performed. For the heteronuclear cluster [YAlO_2_]^+.^, the *C*_2*v*_-symmetric structure **1** (Figure 2 a) corresponds to the global minimum. In **1**, the spin is mainly located at the aluminum atom (0.87 *μ*_B_), while in the homonuclear *D*_2*h*_-symmetric cluster [Y(μ-O)_2_Y]^+.^ (**9**; Figure 3 a), the unpaired electron is delocalized equally over the two bridging yttrium atoms. In the ion/molecule reaction of **1** with N_2_O the isomers **2** and **3** are energetically accessible; the former and more stable one corresponds to an end-on coordination of N_2_O to the Y atom of the cluster, while in **3** the ligand interacts with the aluminum atom. This thermochemical preference of **2** over **3** is mostly due to an electrostatic effect favoring coordination of the incoming nucleophilic N_2_O ligand to the much more positively charged yttrium site of the [YAlO_2_]^+.^ cluster ion (Figure 4). In contrast, in the generation of **3**, the interaction of N_2_O with the aluminum atom is rather weak. Both isomers **2** and **3** are connected by the transition structure **TS 2**/**3**, which is located energetically just below the entrance channel of the separated reactants (Figure 2 a). Direct loss of N_2_ from **2** requires an activation energy of 58 kJ mol^−1^, relative to the entrance channel, and gives rise to [Al(μ-O)_2_Y-O_t_]^+.^ (structure not shown in Figure 2), which is 192 kJ mol^−1^ higher in energy than the product ion [Y(μ-O)_2_Al-O_t_]^+.^ (**5**). Thus, it is not involved in the oxidation of the cluster ion, in which the spin is located exclusively on the oxygen atom of the Al-O_*t*_ moiety (*μ*_B_=1.0). In the formation of **5**, the spin transfer occurs only in the step **3**→**TS 3**/**4**→**4**; in **3**, the spin is still largely located at the aluminum atom.

**Figure 2:**
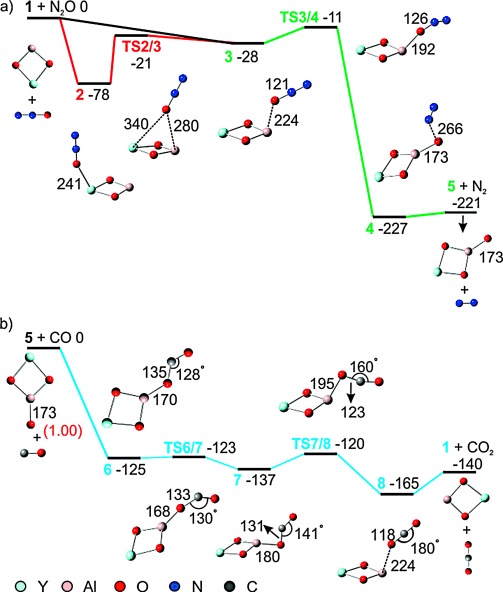
Simplified potential-energy profiles for the reactions of a) [YAlO_2_]^+.^ with N_2_O and b) [YAlO_3_]^+.^ with CO. The energies (given in kJ mol^−1^) are relative to the entrance channel. Some key bond lengths are given in pm, and the Mulliken spin density values (in *μ*_B_) in parentheses.

**Figure 3:**
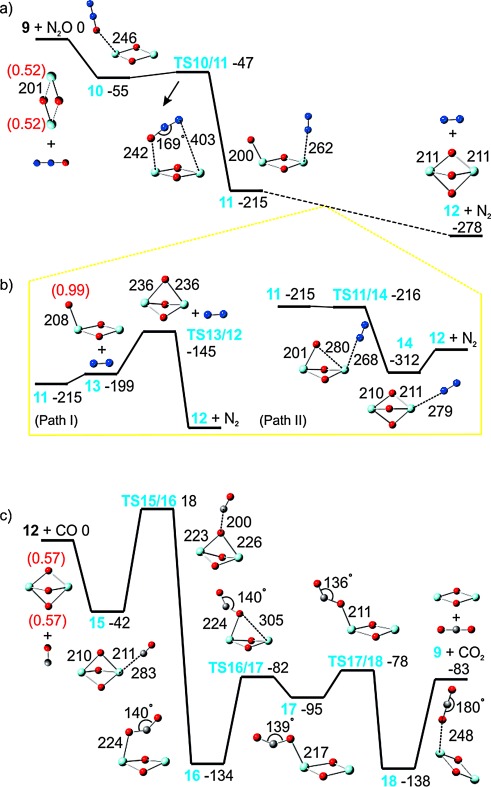
Simplified potential-energy profiles for the reactions of a) Y_2_O_2_^+.^ with N_2_O and c) Y_2_O_3_^+.^ with CO. Details of the reaction sequences from 11 to 12 are shown in (b). The energies (given in kJ mol^−1^) are relative to the entrance channel. Some key bond lengths are given in pm. The Mulliken spin density values (in *μ*_B_) of [Y(μ-O)_2_Y]^+.^ (9), [Y(μ-O)_3_Y]^+.^ (12), and [Y(μ-O)_2_YO_t_]^+.^ (13) are given in parentheses.

**Figure 4:**
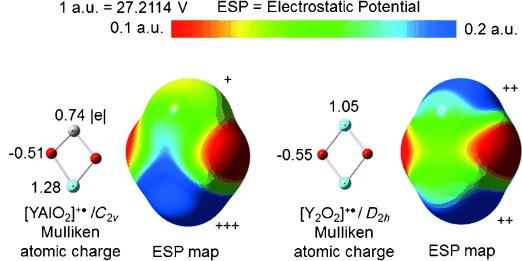
Mulliken atomic charge distributions and electrostatic potentials of the [YAlO_2_]^+.^ (1) and [Y_2_O_2_]^+.^ (9) cluster ions.

As the crucial **TS 3**/**4** is located only 11 kJ mol^−1^ below the entrance channel, the thermochemically rather favored oxidation step [YAlO_2_]^+.^ + N_2_O→[YAlO_3_]^+.^ + N_2_ is kinetically impeded, and thus results in a low reaction efficiency at room temperature. Since the computational accuracy may not be higher than 10 kJ mol^−1^,^13^ **TS 3**/**4** might be relatively close to the entrance channel—in line with the experimental results.

In contrast, the exothermic reduction of [YAlO_3_]^+.^ (**5**) with CO is not hampered by intrinsic energy barriers. However, compared with the rather straightforward reduction of [AlVO_4_]^+.^ by CO,^8^ the potential-energy surface (PES) for the couple **5**/CO is mechanistically more subtle with regard to the liberation of CO_2_ from the encounter complex **6**.

A completely different scenario holds true for the homonuclear [Y_2_O_2_]^+.^ cluster in its reaction with N_2_O. As shown in Figure 3 a, the initially formed end-on encounter complex **10** rearranges through intramolecular N_2_ transfer to the less-coordinated Y atom to produce **11**. While direct liberation of N_2_ gives rise to [Y(μ-O)_2_Y-O_t_]^+.^ (**13**), this path (Figure 3 b, Path I) cannot compete kinetically and thermochemically with the dissociative rearrangement proceeding through **11**→**TS 11**/**14**→**14**→**12** + N_2_ (Figure 3 b, Path II). In line with previous calculations,^14^ the triply oxygen-bridged, *C*_2*v*_-symmetric cluster **12** corresponds to the global minimum; in **12** the spin is delocalized equally over two of the three oxygen atoms of [Y(μ-O)_3_Y]^+.^. For the heteronuclear [YAlO_3_]^+.^ cluster, a structure related to **12** has also been located on the PES; however, as this species is 110 kJ mol^−1^ higher in energy than **5**, it is unlikely to be involved in the course of the catalytic cycle.

While the formation of [Y_2_O_3_]^+.^ from [Y_2_O_2_]^+.^/N_2_O is favored both kinetically and thermochemically, reduction of the former by CO is kinetically impeded by an intrinsic energy barrier (Figure 3 c). Thus, in keeping with the experimental findings, despite an exothermicity of 83 kJ mol^−1^, regeneration of [Y_2_O_2_]^+.^ does not take place and the catalytic cycle cannot be closed.

The distinctly different reactivity of [Y_2_O_3_]^+.^ versus [YAlO_3_]^+.^ in their reactions with CO is just another example of the role spin states often play in chemical reactions.^11^, ^15^ In [YAlO_3_]^+.^ (**5**), where the spin is located at the oxygen atom of the terminal Al-O_t_ unit, there is no need to generate a “prepared state”; consequently, the reaction is barrier-free. In contrast, in [Y_2_O_3_]^+.^ (**12**), the spin is delocalized over two bridging oxygen atoms, thus lacking a prepared state and resulting in an energy barrier for oxygen-atom transfer.

In conclusion, the unexpectedly low catalytic activities of the structurally related cluster ions [YAlO_*n*_]^+.^ and [Y_2_O_*n*_]^+.^ (*n*=2, 3) in their redox reactions with N_2_O/CO have entirely different kinetic origins. While all the individual redox steps fulfill the thermochemical criterion of thermal catalytic oxygen-atom transfer processes, with oxygen-atom affinities (*OA*s) of the clusters located between *OA*(N_2_)=164 kJ mol^−1^ and *OA*(CO)=521 kJ mol^−1^,^[16]^ it is the oxidation with N_2_O that constitutes the bottleneck for the heteronuclear cluster [YAlO_2_]^+.^; in contrast, oxidation of the homonuclear cluster [Y_2_O_2_]^+.^ with N_2_O is possible, but reduction of [Y_2_O_3_]^+.^ by CO in this system is prevented by an energy barrier. These differences are caused by “doping” effects which can control the local charge and spin distributions in the reduction of N_2_O and the oxidation of CO, respectively.^17^ In a more general sense, the reactivities of heteronuclear oxide cluster ions, in comparison with their homonuclear counterparts, can be increased,9f decreased,^17^ not significantly affected,9c or in some cases even the product distributions can be altered,^18^ and these observations highlight the potential to control chemical processes by selective cluster doping.

## Experimental and Computational Section

All experiments were performed with a Spectrospin CMS 47X Fourier-transform ion-cyclotron resonance (FTICR) mass spectrometer equipped with an external ion source, as described elsewhere.^19^ In brief, the cluster cations [YAlO_2_]^+.^ and [Y_2_O_2_]^+.^ were generated by laser ablation of an yttrium/aluminum target (with a molar ratio of 1:1) by using a Nd:YAG laser operating at 1064 nm in the presence of 0.5 % O_2_, seeded in a helium carrier gas; [YAlO_3_]^+.^ cannot be generated directly with this experimental setup. By using a series of potentials and ion lenses, the ions were transferred into the ICR cell, which was positioned in the bore of a 7.05 T superconducting magnet. After collisional thermalization by pulses of argon (ca. 2×10^−6^ mbar), the [YAlO_2_]^+.^, [Y_2_O_2_]^+.^, and [Y_2_O_3_]^+.^ ions were mass-selected and studied with respect to their reactions by introducing the substrates N_2_O (or CO) through a leak-valve in the ICR cell. The experimental second-order bimolecular rate coefficients at room temperature were evaluated by assuming a pseudo-first-order kinetic approximation after calibration of the measured pressure and consideration of the ion-gauge sensitivities. The bimolecular rate coefficients have an uncertainty of ±30 %.^20^ A temperature of 298 K was assumed for the thermalized cluster ions.^20^

The density functional theory (DFT) calculations were carried out using the Gaussian 09 program^21^ employing the hybrid B3LYP exchange-correlation functional.^22^ The TZVP basis sets^23^ were used for C, N, O, and Al, and the polarized triple-ζ valence basis sets (Def2-TZVP)^24^ were selected for Y. Geometry optimizations with full relaxation of all atoms were performed. Vibrational frequency calculations were carried out to check that the reaction intermediates have zero imaginary frequency. The energies (given in kJ mol^−1^) were corrected by zero-point vibrational energy (ZPE) contributions. Intrinsic reaction-coordinate calculations^25^ were performed to connect the TSs with local minima.

## 

Dedicated to Professor Peter B. Armentrout on the occasion of his 60th birthday

## References

[1] 2009 U.S. Greenhouse Gas Inventory Report, Environmental Protection Agency http://tinyurl.com/emissionsreport.

[2a] Prather MJ (1998). Science.

[2b] Robertson A, Overpeck J, Rind D, Mosley-Thompson E, Zielinski G, Lean J, Koch D, Penner J, Tegen I, Healy R (2001). J. Geophys. Res.

[2c] Ravishankara AR, Daniel JS, Portmann RW (2009). Science.

[3] Freund H-J, Meijer G, Scheffler M, Schlögl R, Wolf M (2011). Angew. Chem.

[4] Kappes MM, Staley RH (1981). J. Am. Chem. Soc.

[5a] Baranov V, Javahery G, Hopkinson AC, Bohme DK (1995). J. Am. Chem. Soc.

[5b] Kretzschmar I, Fiedler A, Harvey JN, Schröder D, Schwarz H (1997). J. Phys. Chem. A.

[5c] Brönstrup M, Schröder D, Kretzschmar I, Schwarz H, Harvey JN (2001). J. Am. Chem. Soc.

[5d] Koyanagi GK, Bohme DK (2001). J. Phys. Chem. A.

[5e] Lavrov VV, Blagojevic V, Koyanagi GK, Orlova G, Bohme DK (2004). J. Phys. Chem. A.

[5f] Blagojevic V, Orlova G, Bohme DK (2005). J. Am. Chem. Soc.

[5g] Schlangen M, Schwarz H (2012). Cat. Lett.

[6a] Achatz U, Berg C, Joos S, Fox BS, Beyer MK, Niedner-Schatteburg G, Bondybey VE (2000). Chem. Phys. Lett.

[6b] Balaj OP, Balteanu I, Roßteuscher TTJ, Beyer MK, Bondybey VE (2004). Angew. Chem.

[6c] Balteanu I, Balaj OP, Beyer MK, Bondybey VE (2004). Phys. Chem. Chem. Phys.

[6d] Lv L, Wang Y-C, Jin Y (2011). Theor. Chem. Acc.

[7a] Taylor HS (1925). Proc. R. Soc. London Ser. A.

[7b] Schwab GM, Pletsch E (1929). Z. Phys. Chem.

[7c] Davis RJ (2003). Science.

[7d] Horn K (2004). Science.

[7e] Thomas JM, Raja R, Lewis DW (2005). Angew. Chem.

[7f] Somorjai GA, Park JY (2008). Angew. Chem.

[7g] Ertl G (2008). Angew. Chem.

[7h] Lang S, Bernhardt TM (2012). Phys. Chem. Chem. Phys.

[7i] Behrens M, Studt F, Kasatkin I, Kühl S, Hävecker M, Abild-Petersen F, Zander S, Girgsdies F, Kurr P, Kniep B-L, Tovar M, Fischer RW, Nørskov JK, Schlögl R (2012). Science.

[8] Wang Z-C, Dietl N, Kretschmer R, Weiske T, Schlangen M, Schwarz H (2011). Angew. Chem.

[9a] Johnson GE, Mitrić R, Tyo EC, Bonačić-Koutecký V, Castleman AW (2008). J. Am. Chem. Soc.

[9b] Johnson GE, Mitrić R, Bonačić-Koutecký V, Castleman AW (2009). Chem. Phys. Lett.

[9c] Nößler M, Mitrić R, Bonačić-Koutecký V, Johnson GE, Tyo EC, Castleman AW (2010). Angew. Chem.

[9d] Tyo EC, Nößler M, Mitrić R, Bonačić-Koutecký V, Castleman AW (2011). Phys. Chem. Chem. Phys.

[9e] Castleman AW (2011). Catal. Lett.

[9f] Ma J-B, Wang Z-C, Schlangen M, He S-G, Schwarz H (2012). Angew. Chem.

[10] Su T, Bowers MT (1973). Int. J. Mass Spectrom. Ion Phys.

[11] Dietl N, Schlangen M, Schwarz H (2012). Angew. Chem.

[12] Pyun CW (1971). J. Chem. Educ.

[13] Koch W, Holthausen MC (2000). A Chemist’s Guide to Density Functional Theory.

[14] Zhao Y-X, Ding X-L, Ma Y-P, Wang Z-C, He S-G (2010). Theor. Chem. Acc.

[15a] Wu X-N, Zhao Y-X, Xue W, Wang Z-C, He S-G, Ding X-L (2010). Phys. Chem. Chem. Phys.

[15b] Wu X-N, Ding X-L, Bai S-M, Xu B, He S-G, Shi Q (2011). J. Phys. Chem. C.

[15c] Lai W, Li C, Chen H, Shaik S (2012). Angew. Chem.

[16] Böhme DK, Schwarz H (2005). Angew. Chem.

[17a] Li Z-Y, Zhao Y-X, Wu X-N, Ding X-L, He S-G (2011). Chem. Eur. J.

[17b] Ding X-L, Wu X-N, Zhao YX, He S-G (2012). Acc. Chem. Res.

[18a] Koszinowski K, Schröder D, Schwarz H (2003). J. Am. Chem. Soc.

[18b] Koszinowski K, Schröder D, Schwarz H (2003). ChemPhysChem.

[18c] Dietl N, Höckendorf RF, Schlangen M, Lerch M, Beyer MK, Schwarz H (2011). Angew. Chem.

[19a] Eller K, Schwarz H (1989). Int. J. Mass Spectrom. Ion Processes.

[19b] Eller K, Zummack W, Schwarz H (1990). J. Am. Chem. Soc.

[19c] Engeser M, Weiske T, Schröder D, Schwarz H (2003). J. Phys. Chem. A.

[20] Schröder D, Schwarz H, Clemmer DE, Chen YM, Armentrout PB, Baranov VI, Bohme DK (1997). Int. J. Mass Spectrom. Ion Processes.

[21] Frisch MJ

[22a] Lee CT, Yang WT, Parr RG (1988). Phys. Rev. B.

[22b] Becke AD (1988). Phys. Rev. A.

[22c] Becke AD (1993). J. Chem. Phys.

[23] Schäfer A, Huber C, Ahlrichs R (1994). J. Chem. Phys.

[24a] Andrae D, Häußermann U, Dolg M, Stoll H, Preuß H (1990). Theor. Chim. Acta.

[24b] Weigend F, Ahlrichs R (2005). Phys. Chem. Chem. Phys.

[25a] Fukui K (1970). J. Phys. Chem.

[25b] Fukui K (1981). Acc. Chem. Res.

[25c] Gonzalez C, Schlegel HB (1989). J. Chem. Phys.

[25d] Gonzalez C, Schlegel HB (1990). J. Phys. Chem.

[25e] Truhlar DG, Gordon MS (1990). Science.

